# Shifts in Soil Bacterial Diversity and Abundance at Various Taxonomic Levels in Tropical Peatlands Under Land‐Use Change

**DOI:** 10.1155/ijm/3336189

**Published:** 2026-08-29

**Authors:** Delita Zul, Funny Aicha Sharin Saragih, Putri Kristina Zebua, Nelvia

**Affiliations:** ^1^ Department of Biology, Faculty of Mathematics and Natural Sciences, University of Riau, Pekanbaru, Indonesia, unri.ac.id; ^2^ Center for Peatland and Disaster Studies, University of Riau, Pekanbaru, Indonesia, unri.ac.id; ^3^ Department of Agrotechnology, Faculty of Agriculture, University of Riau, Pekanbaru, Indonesia, unri.ac.id

**Keywords:** 16S rRNA gene, fire, land-use, soil bacteria, tropical peatland

## Abstract

Tropical peatlands are among the largest global carbon sinks and face increasing impacts from drainage, fire, and land‐use change. These disturbances modify soil physicochemical characteristics and influence bacterial community structure. However, the response of bacterial communities to these changes is incompletely understood. This study examined bacterial community composition at six peatland sites in the Giam Siak Kecil–Bukit Batu Biosphere Reserve, Riau, Indonesia. The sites represent various land‐use types: secondary forest, restored peat, burned peat, oil palm, rubber, and acacia plantations. The analysis employed 16S rRNA gene sequencing combined with measurements of soil physicochemical properties. Results showed that key bacterial phyla, such as Acidobacteriota, Pseudomonadota, and Actinomycetota, were consistently found across sites, though their relative abundances varied. Burned sites had increased abundances of Bacillota and other copiotrophic groups and decreased abundances of several oligotrophic taxa. Beta diversity analysis showed differences in community patterns among sites, but multivariate analysis did not show significant differences between land‐use types. Variation in bacterial community composition is associated with soil pH and nutrient availability, including available phosphorus, exchangeable potassium, and exchangeable magnesium, although these relationships are not always statistically supported. A small number of ASVs were detected across all sites, suggesting overlapping community members amid high spatial heterogeneity. Overall, tropical peatland bacterial communities form within a narrow environmental framework in which distinctive physicochemical properties constrain which microbes can survive. Land‐use changes are primarily associated with shifts in the relative abundance of bacterial taxa rather than complete community turnover. Given the limited number of samples and single time‐point observations, these findings are exploratory and provide a basis for further research.

## 1. Introduction

Peatlands play a vital role in the global carbon cycle and in regulating climate [1, 2]. Peatlands comprise 2.8%–3% of the global land area [3], yet they sequester over 30% of the world′s soil carbon as organic matter, which accumulates under waterlogging and low‐oxygen conditions [4, 5]. The majority of tropical peatlands are located in Southeast Asia, with Indonesia possessing the largest area of tropical peat globally [6, 7], where they have been increasingly affected by logging, drainage, fires, and conversion to plantation (PL) systems such as oil palm, acacia, and rubber [2, 5, 8–10]. These disturbances modify the physicochemical properties of peat soils [11–14], including water table depth [15], aeration, pH, and nutrient availability [16–18]. As a result, peat soils become drier, more aerobic, and less acidic, with altered nutrient profiles compared to undisturbed peat [19–21]. Thus, these alterations in environmental conditions are linked to changes in soil microbial community, which are crucial for decomposition and nutrient cycling in peat ecosystems [22, 23].

Peat soils display unique environmental conditions with strong ecological stresses. Persistent acidity, high organic carbon (Org‐C) content, and low nutrient availability limit the range of microorganisms that can survive [22, 24, 25]. In this context, bacterial communities in peat soils are often structured along an oligotrophic–copiotrophic gradient [1, 26, 27]. Intact peatlands are typically dominated by oligotrophic and acid‐tolerant bacteria [28, 29]. Here, carbon sources are complex and poorly degradable, and oxygen availability is limited. As a result, bacterial groups such as Acidobacteriota often dominate in natural peat soils [24]. However, when peatlands are drained, burned, or converted to PLs, their environmental conditions change. Increased aeration, rising nutrient availability, and changes in vegetation and litter quality make carbon inputs more readily decomposable [12, 30]. Under such conditions, copiotrophic bacteria such as Pseudomonadota and Actinomycetota become more abundant [31]. In more disturbed environments, especially after fires, disturbance‐tolerant and spore‐forming bacteria, such as members of the Bacillota, show a significant increase in relative abundance. This oligotroph–copiotroph framework has been widely used to explain microbial community patterns in soils [31–33], particularly in temperate peat ecosystem [34].

Despite the growing number of studies on tropical peat microbiomes, several key questions remain unresolved. Many existing studies in Southeast Asia have focused on a single land‐use type, such as peat swamp forest, oil palm plantation (OPP), or burned peatland [11, 22, 35, 36]. The limited scope of these studies makes it difficult to understand how microbial communities change across land‐use gradients within the same peat landscape. Additionally, soil physicochemical properties, vegetation changes, and microbial community composition are often examined separately [16, 37]. Few studies consider these aspects in a unified ecological framework [38, 39]. As a result, our understanding of how environmental filtering, resource availability, and disturbance history interact to shape peat microbial communities remains incomplete. This knowledge gap is particularly pronounced in tropical peatlands affected by drainage and fire, where environmental conditions vary markedly across the landscape. A key question is whether land‐use change leads to complete taxonomic turnover in tropical peatlands or primarily alters the relative abundance of existing bacterial groups. Disturbances may not entirely replace microbial communities but may instead reorganize the abundance within a persistent core microbiome across land‐use types. Addressing this issue requires an integrative, landscape‐scale approach that simultaneously examines microbial communities and soil physicochemical properties across different peatland‐use types.

To address this question, we compared bacterial communities across peatland land‐use types within the Giam Siak Kecil–Bukit Batu (GSK‐BB) Biosphere Reserve, Riau, Indonesia. By combining high‐resolution 16S rRNA gene sequencing with soil physicochemical properties, this study examines how bacterial communities vary along a gradient of land‐use disturbance while identifying the extent to which a conserved peat‐associated core microbiome persists across sites. This landscape encompasses a gradient ranging from secondary peat forest to restored peat, burned peat, and peatlands converted to oil palm, rubber, and acacia plantations (APs). All study sites are located within the same peatland area but differ in disturbance history, vegetation type, and management practices [40, 41]. By studying different types of land‐use within the same landscape, the influence of large‐scale climatic and geological factors can be minimized, allowing differences in bacterial communities to be more closely linked to land‐use differences and the physicochemical conditions of the peat soil.

Based on the concept of environmental filtering in peat soils, it is predicted that relatively intact or lightly disturbed peat sites will be dominated by higher relative abundances of oligotrophic and acidophilic bacterial groups. Conversely, peatlands experiencing drainage, land conversion, or fire are expected to show an increased relative abundance of copiotrophic and disturbance‐tolerant bacterial groups. However, given that peat soils are characterized by persistent environmental constraints, such as high acidity and high Org‐C content, we hypothesize that land‐use changes are not always accompanied by a complete turnover of taxa, but rather by changes in the relative abundance of dominant bacterial groups. Therefore, the core community of peat bacteria is expected to remain present across land‐use types. Therefore, the objectives of this study were to (i) characterize the bacterial community composition across different types of tropical peatland use, (ii) analyze differences in bacterial community diversity and composition across locations and land‐use groups, and (iii) evaluate the relationship between soil physicochemical properties and bacterial community structure across peatland‐use gradients.

## 2. Materials and Methods

### 2.1. Site Description and Soil Sampling Method

**Figure 1:**
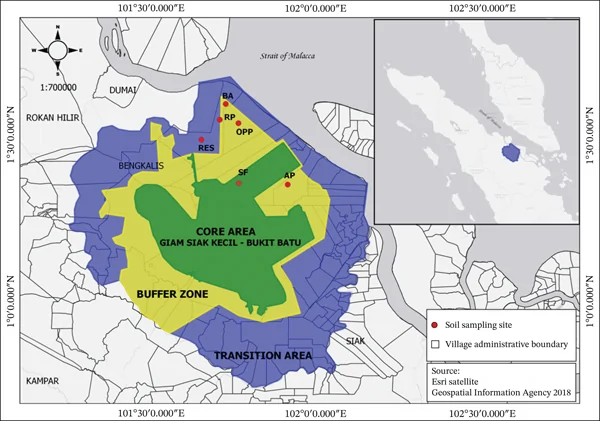
Map of the six tropical peatlands representing different land‐use types. Abbreviations: SF, secondary forest; RES, restored peat; BA, burnt area; OPP, oil palm plantation; RP, rubber plantation; and AP, acacia plantation.

Peat soil samples were collected from the GSK‐BB Biosphere Reserve, Bengkalis Regency, Riau Province, Indonesia (Figure 1). The GSK‐BB Biosphere Reserve is one of 21 biosphere reserves in Indonesia. The GSK‐BB biosphere reserve is a peat swamp ecosystem spanning 705,271 ha across Bengkalis Regency, Siak Regency, and Dumai City, comprising 23 subdistricts and 148 villages. The reserve is managed under a zoning system consisting of a core zone (178,722 ha), a buffer zone (222,426 ha), and a transition zone (304,123 ha). The core zone includes the Giam Siak Kecil Wildlife Sanctuary (84,967 ha), Bukit Batu Wildlife Sanctuary (21,500 ha), and 72,255 ha of conservation forest. Meanwhile, the buffer zone, covering 222,426 ha, functions as a protective area surrounding the core zone and has largely been converted to industrial PLs and production forests. The transition zone, spanning 304,123 ha, is the largest outermost region to have undergone conversion to OPPs, agricultural land, industrial tree PLs, and community settlements [40, 41]. This zoning system was established to balance biodiversity conservation with sustainable land‐use practices and local economic development.

Peat soil samples were collected from six representative land‐use types, namely OPPs, rubber plantations (RPs), APs, restored peat sites (RESs), burnt areas (BAs), and secondary forest (SF) as a reference site. At each site, four field subsamples were collected. These subsamples were analyzed individually for soil physicochemical properties but were pooled in equal proportions to generate a single composite sample for 16S rRNA gene sequencing. Consequently, bacterial community analyses were based on six composite sequencing samples (one per site) and represent site‐level composite profiles rather than replicated site‐level measurements. For broader statistical comparison, sites were grouped into PL (OPP, RP, and AP) and nonplantation (NPL: SF, RES, and BA) categories. A detailed description of each peat soil site sampled is presented in Table 1. Previous studies within the same peat dome system reported shallower water table depths at the SF site (~11–12 cm) than at restored, burned, and PL sites (~61–78 cm) [42, 43]. The region receives an average monthly rainfall of approximately 218 mm.

**Table 1:** Description of peat soil sampling sites.

Soil sampling site	Sample group	Coordinate	Description
Secondary forest (SF)	NPL	N 01°26 ^′^13,68 ^″^	Located in the core zone, with a peat thickness of 9 m estimated. The soil sampling location is approximately 100 m away from the forest edge. The site′s vegetation consists of native peat plants, such as *Litsea* sp., *Palaquium* sp., *Shorea* sp., *Campnosperma* sp., and *Cinnamomum* sp.
		E 101°51 ^′^11,1″	
Restored peat (RES)	NPL	N 01°38 ^′^29,4 ^″^	Located in the transition zone, the land is a former oil palm plantation that was frequently flooded and burned in 2008. Restoration began in 2011 with the planting of native peat plants, including *Swietenia* sp., *Macaranga* sp., *Dyera costulata*, *Payena* sp., *Alstonia* sp., *Hevea brasiliensis*, *Nicotiana* sp., *Aquilaria* sp., *Vatica* sp., *Rhizophora* sp., and *Syzygium* sp. The blocking channel was built in 2015.
		E 101°44 ^′^19,9 ^″^	
Burnt area (BA)	NPL	N 01°36 ^′^33,8 ^″^	Located in the buffer zone, the peat layer is approximately 12 m thick and characterized by grass‐dominated vegetation. Peat samples were obtained approximately 1 month after the site was burned.
		E 101°42 ^′^13,0 ^″^	
Oil palm plantations (OPP)	PL	N 01°28 ^′^45,4 ^″^	The plantation is located in the buffer zone and has never been burned. The primary vegetation consists of 11‐year‐old oil palms. Other types of vegetation identified include lianas, ferns, and weeds.
		E 101°55 ^′^05,6 ^″^	
Rubber plantations (RP)	PL	N 01°38 ^′^07,0 ^″^	This rubber plantation is located in the buffer zone and is community‐owned. The plantation is poorly maintained, and the rubber trees are approximately 6‐7 years old. Other vegetation found is lianas, ferns, and weeds.
		E 101°44 ^′^02,7 ^″^	
Acacia plantation (AP)	PL	N 01°29 ^′^43,3 ^″^	Located in the buffer zone and managed by a private company, the peat thickness is ±9 m. The land is planted with 4‐year‐old *Acacia crassicarpa*, in rotation three. The land is surrounded by canals built in 2016. Fertilizer is applied to the acacia plants only when they are 1 year old and is not reapplied until the pulpwood is harvested. The forest floor is covered with lianas and ferns and has never been burned.
		E 101°47 ^′^47,0 ^″^	

Abbreviations: NPL, nonplantation; PL, plantation.

Soil sampling was conducted using purposive sampling to represent the dominant peatland land‐use types within a relatively uniform peat dome landscape. Four subsampling points were established approximately 50 m apart along transects at each site. Sampling points were located on relatively flat peat surfaces while avoiding hummock–hollow transitions to minimize microtopographic and maintain comparable microenvironmental conditions. Vegetation age, canopy structure, and surface moisture were relatively uniform among subsampling points within each site. Surface litter was removed prior to sampling. Soil for physicochemical analysis was collected with a shovel following the method of Syaufina and Hamzah [44] and immediately placed in tightly sealed plastic bags. Samples for bacterial community analysis were collected using a soil ring core (5 cm diameter × 40 cm height) [45]. Separate ring cores were used for each site to prevent cross‐contamination. Soil samples were collected from the surface peat layer at a depth of 0–30 cm, representing the biologically active acrotelm, and all sites were sampled during the same period. A total of 24 field subsamples were collected across the six study sites. Samples for physicochemical analyses were stored at room temperature, whereas soil samples intended for DNA extraction were stored at −20°C until further processing.

### 2.2. Soil Physicochemical Characterization

The physicochemical characteristics measured included soil temperature, pH, and nutrient content. Soil temperature was measured using the temperature probe of a handheld four‐in‐one soil survey instrument (iTuin) by inserting it into the soil, and the results were recorded. Soil pH was measured by dipping the pH meter electrode directly into the soil slurry, a mixture of soil and distilled water at a 1:2.5 (w/v) ratio, as previously described [46]. This approach allows pH measurements that reflect representative field conditions. The loss‐on‐ignition (LOI) method, as previously described by Nelson and Sommers [47], was used to quantify soil Org‐C. Soil samples were dried and then combusted in a furnace at approximately 550°C for 6 hours [48]. The resulting mass loss is attributed to the loss of burnt organic matter, allowing the percentage of Org‐C to be calculated. Total nitrogen (N‐total) was determined by the Kjeldahl method [49], in which the soil is digested with concentrated sulfuric acid and a metal catalyst to convert organic nitrogen to ammonium ions, which are then measured by distillation and titration (FOSS Kjeltec). For available phosphorus (P‐avail), the Bray I method [50], suitable for acid soils, was employed by extracting the soil with a solution of 0.03 M hydrochloric acid and 0.025 M hydrofluoric acid. The phosphate in this extract solution is then analyzed colorimetrically using a molybdate reagent and a spectrophotometer. Exchangeable potassium (K‐exch) and exchangeable magnesium (Mg‐exch) were determined using the ion‐exchange resin method [51], which involves the use of a cation‐exchange resin to adsorb ions from a soil slurry. K‐exch was measured using a Jenway PFP7 flame photometer, whereas Mg‐exch was analyzed using a Thermo Scientific iCE 3000 atomic absorption spectrophotometer.

### 2.3. DNA Extraction

Soil DNA was extracted using the CTAB/SDS method, as described previously [52]. Briefly, 5 g of soil was mixed with 10 mL of extraction buffer (100 mM Tris/HCl [pH 8.0], 100 mM EDTA [pH 8.0], 100 mM sodium phosphate buffer [pH 8.0], 1.5 M NaCl, 1% [w/v] CTAB, 100 mM CaCl2, 10 mg proteinase K/mL, and 10 mg lysozyme/mL) in Oakridge tubes and incubated at 37°C for 1 h with shaking at 200 rpm. After adding 2 mL of 20% (w/v) SDS, the mixture was incubated in a water bath at 65°C for 2 h, with mixing by inversion every 10–15 min. The tube was centrifuged at 7000 g for 20 min at 4°C. The pellets were incubated for 15 min at 65°C in 4.5 mL of extraction buffer and 0.5 mL of 20% (w/v) SDS. The supernatants of the three extractions were collected and mixed with equal volumes of chloroform and isoamyl alcohol (24:1, v/v). To collect the upper aqueous phase, the tube was centrifuged again at 14,000 g for 20 min at 4°C. After precipitation of the DNA pellet, 0.1 volume of 3 M sodium acetate and 0.4 volume of 30% (w/v) PEG‐8000 (polyethylene glycol) were added to the aqueous phase and incubated at −20°C. After 2 h, the DNA pellets were centrifuged at 14,000 g for 15 min at 4°C, then washed once with 70% (v/v) ethanol at room temperature and dried in air. The dry pellet was dissolved in 1 mL of TE buffer, then mixed with an equal volume of chloroform/isoamyl alcohol (24:1, v/v). The aqueous phase was collected by centrifugation at 14,000 g for 15 min at 4°C, and the DNA was precipitated with 0.7 volumes of isopropanol and incubated overnight at −20°C. The DNA pellets were centrifuged at 14,000 g for 15 min at 4°C. The pellets were washed with 5 M NaCl, soaked in 70% (v/v) ethanol, and air dried. The pellet was dissolved in 200 *μ*L of 1x TE buffer. DNA concentration and integrity were determined using 1% agarose gel electrophoresis. DNA purity was evaluated spectrophotometrically (NanoDrop, Thermo Scientific, United States) by measuring the A260/280 ratio to assess protein contamination and the A260/230 ratio to detect humic contamination. According to the manufacturer′s instructions, the DNA was diluted to 1 ng/*μ*L in sterile water. The extracted soil DNA was stored at −20°C.

### 2.4. Amplification of 16S rRNA Gene and Purification of PCR Products

Amplification of the 16S rRNA gene was performed in the V3‐V4 region using specific primers 338F (5 ^′^‐ACTCCTACGGGAGGCAGCA‐3 ^′^) and 806R (5 ^′^‐GGACTACHVGGGTWTCTAAT‐3 ^′^) [53]. Polymerase chain reaction (PCR) was performed in triplicate using the following cocktail: 4 *μ*L of 5x Phusion HF Buffer, 0.4 *μ*L of 10 mM dNTPs, 1 *μ*L of each primer sample (0.2 M), 0.2 *μ*L of Phusion DNA Polymerase, and 0.08 *μ*L (0.02 ng/mL) of DNA template. PCR cocktails were prepared according to the manufacturer′s instructions using the Phusion High‐Fidelity PCR Master Mix Kit (New England Biolabs). Amplification conditions were as follows: (1) initial denaturation at 98°C for 1 min; (2) 25 cycles of denaturation at 98°C for 5 s, annealing at 60°C for 15 s, and extension at 72°C for 2 min 30 s; (3) final extension at 72°C for 10 min; and (4) hold at 4°C. The same volume of 1x loading buffer (containing SYBR Green) was added to the PCR product, and the mixture was electrophoresed on a 2% agarose gel to visualize the PCR product. Samples with bright bands between 400 and 450 bp were selected for further experiments. The PCR products were then mixed in an equidensity ratio. The resulting combined product was then purified using the Qiagen Gel Extraction Kit according to the manufacturer′s protocol (Qiagen, Germany).

### 2.5. Library Preparation and Sequencing

Purified 16S rRNA gene amplicons were used to prepare sequencing libraries using the NEBNext Ultra DNA Library Prep Kit for Illumina (New England Biolabs, United States) according to the manufacturer′s protocol. PCR amplicons were end‐repaired and dA‐tailed to add 3 ^′^‐adenine overhangs, enabling ligation of Illumina sequencing adapters. Adapter‐ligated fragments were indexed and PCR‐amplified to generate the final library. Library size and quality were assessed using an Agilent 2100 Bioanalyzer (Agilent Technologies, United States). Electropherogram profiles confirmed the expected size. Library concentration was measured using a Qubit 2.0 Fluorometer (Thermo Fisher Scientific, United States) and validated by quantitative PCR (qPCR) prior to sequencing. Following the manufacturer’s protocol, we used an Illumina HiSeq 2500 platform to sequence the DNA libraries. Paired‐end sequencing was used to read both ends of the DNA fragment simultaneously, with a read length of 250 bp per end. Library preparation and DNA sequencing were performed by Novogen Co. Ltd, a genomic analysis service provider, through its representative PT. Genetika Science Indonesia is based in Jakarta, Indonesia.

### 2.6. Statistical and Bioinformatics Analyses

#### 2.6.1. Soil Physicochemical Statistical Analyses

Soil physicochemical properties were analyzed using one‐way analysis of variance (ANOVA) to assess significant differences among soil sampling sites. When ANOVA results were significant (*p* < 0.05), Duncan′s Multiple Range Test (DMRT) was applied as a post hoc test using SPSS software Version 22.0 (IBM Corp., United States).

#### 2.6.2. 16S rRNA Gene Sequence Analysis Using QIIME2 (Quantitative Insights Into Microbial Ecology 2)

Complete amplicon fragments were analyzed using QIIME2 software Version 2025.7 [54], which provides integrated tools for sequence preprocessing, denoising, and taxonomic classification. Raw FASTQ data were first assessed using FastQC to evaluate sequence quality and read length distribution. Then, adapter and primer sequences were removed using the Cutadapt plugin, retaining only the target region of the 16S rRNA gene for later analyses [55]. Sequence denoising, quality filtering, chimera removal, and interference from amplicon sequence variants (ASVs) were performed using the DADA2 plugin in QIIME2 [56]. These steps included eliminating low‐quality sequences, trimming their ends, and removing sequences that did not meet quality thresholds. The identified ASVs were treated as high‐resolution taxonomic units, and an ASV feature table was created to quantify the number of sequences per sample. Taxonomic classification was performed using a Naïve Bayes classifier trained on the SILVA SSU database (release 138.2) for the 16S rRNA V3–V4 gene. Phylogeny‐based analyses involved constructing a phylogenetic tree of representative ASV sequences using the QIIME2 align‐to‐tree‐mafft‐fasttree pipeline. Sequences were aligned using MAFFT, and poorly aligned regions were masked to reduce noise. A phylogenetic tree was then inferred using FastTree and subsequently rooted. The resulting tree was used to calculate phylogenetic beta‐diversity metrics, including weighted and unweighted UniFrac distances. To address differences in sequencing depth, ASV data were rarefied to a common depth before further analysis. This step aimed to equalize sequencing effort across samples and reduce potential bias from differences in read counts. Prior to rarefaction, the sequencing depth of all samples ranged from 39,959 to 73,916 reads (AP = 39,959; BA = 73,916; OPP = 42,622; RES = 52,792; RP = 45,652; SF = 44,671). All samples were then rarefied to 39,000 reads. All samples were above this threshold; no samples were excluded from further analysis. Statistical analyses were performed using R software version 4.5.0 [57] with the vegan package (Version 2.7.2) [58]. Finally, taxonomic profiles were summarized at the phylum, class, order, family, and genus levels.

#### 2.6.3. Abundance, Diversity, and Ordination Analyses

Abundance, diversity, and ordination analyses were performed in R (Version 4.5.0) [57] using the packages phyloseq (Version 1.52.0) [59], vegan (Version 2.7.1) [58], microbiome (Version 1.22.0), and ggplot2 (Version 4.0.0) [60]. All analyses were performed on the ASV‐based taxonomic data that had been filtered and normalized during sequence processing in QIIME2. Taxonomic composition of bacteria at the phylum, class, family, and genus levels was visualized using bar plots and heatmaps. Differences in relative abundance among sites were assessed at these taxonomic levels. To evaluate community similarity, ASV data were converted to presence–absence format, and the Jaccard index was calculated. Pairwise Jaccard similarity was used to compare the SF site with other sites (RES, BA, OPP, RP, and AP). In addition, the proportion of ASVs shared across all six sites was calculated to estimate the extent of a common core community. Unique and shared ASVs within and between samples and groups were visualized using Venn and Flower diagrams. Intersections represent shared ASVs, whereas nonintersecting areas indicate unique ASVs.

Alpha and beta diversity were calculated to evaluate bacterial community structure across land‐use types. Alpha diversity was estimated using Shannon diversity, Simpson index, and the abundance‐based coverage estimator (ACE). Because each site was represented by a single composite sample, statistical comparisons were conducted only at the broader grouping level by comparing PL and NPL sites. Beta diversity was assessed using Bray–Curtis dissimilarity as well as weighted and unweighted UniFrac distances. Bray–Curtis dissimilarity was used to evaluate differences in bacterial community composition based on ASV relative abundances, whereas UniFrac metrics incorporated phylogenetic relationships among taxa. Unweighted UniFrac looked at phylogenetic turnover by considering only the presence or absence of lineages, whereas weighted UniFrac included both phylogenetic relationships and taxon abundance. This method enabled comparisons of community differences across peatland sites from both abundance‐based and phylogenetic perspectives. Ordination patterns were visualized using nonmetric multidimensional scaling (NMDS) based on Bray–Curtis dissimilarity. NMDS convergence and stress values were evaluated to ensure appropriate ordination performance. Differences in community composition between groups were tested using analysis of similarity (ANOSIM) based on beta‐diversity distance matrices, with 999 permutations. This nonparametric permutation test assesses whether community dissimilarities between groups exceed those within groups and yields an R statistic that reflects the degree of group separation.

The relationship between bacterial community composition and soil physicochemical properties was evaluated using distance‐based redundancy analysis (db‐RDA) implemented with the *dbrda* function in the *vegan* package, based on Bray–Curtis dissimilarity. The overall significance of the model and the contribution of each environmental variable were assessed using permutation tests (anova.cca) with 999 permutations. Ordination biplots were generated to visualize the relationships between bacterial communities and soil physicochemical variables. In these biplots, the direction and length of environmental vectors indicate their influence on community structure. Additionally, Spearman′s rank correlation analysis was conducted to assess associations among soil physicochemical properties, bacterial diversity indices, and the relative abundance of dominant bacterial taxa.

## 3. Results

### 3.1. Soil Physicochemical Properties Across the Six Sampling Sites

All soils at each site showed peat characteristics, with very high Org‐C (49%–56%), high C/N ratios (over 43), and very acidic pH values (3.5–5.0) (Table 2). Despite these similarities, nutrient contents differed along the disturbance gradient. Specifically, the burnt area (BA) exhibited significantly higher P‐avail and Mg‐exch than other sites and had relatively high K‐exch. In comparison, PL sites (OPP and AP) had lower exchangeable base cation concentrations, whereas natural (SF) and RES sites maintained intermediate levels. Regarding soil acidity, soil pH varied only slightly among sites, with AP the most acidic and RES the least. N‐total was very high at all sites, with SF having the highest levels. C/N ratios were very high (> 25%) across all sites and did not differ significantly among sites. These results support classifying the peat soil as oligomesotrophic, indicating a low level of available nitrogen despite a high N‐total. The data demonstrate that although the fundamental natural characteristics of the peat remained constant across the study sites, fire disturbance led to significant increases in nutrient and cation levels, thereby distinguishing the BA site from the other sites.

**Table 2:** Soil physicochemical properties of six soil sampling sites.

Properties	Soil sampling site					
	SF	RES	BA	OPP	RP	AP
Temperature (°C)	28.5 ± 0.71a	29 ± 0.00a	29 ± 0.00a	29 ± 0.00a	28.5 0.07a	28 ± 0.00a
pH	3.95 ± 0.07ab	5 ± 0.00c	4.25 ± 0.35b	4.24 ± 0.07b	4.23 ± 0.35b	3.5 ± 0.14a
Org‐C (%)	55.65 ± 0.07bc	54.7 ± 0.00bc	52.2 ± 1.56ab	49.35 ± 3.18a	55.5 ± 0.00bc	56.4 ± 00c
N‐total (%)	1.29 ± 0.06c	0.91 ± 0.00a	0.99 ± 0.11ab	0.93 ± 0.26a	0.98 ± 0.01ab	0.92 ± 0.11a
Ratio C/N	43.18 ± 1.95a	60.11 ± 0.00a	52.85 ± 7.19a	56.08 ± 19.31a	56.64 ± 0.81a	62.06 ± 7.19a
P‐avail (ppm)	31.25 ± 5.16a	27.6 ± 0.00a	439.35 ± 30.48b	36.15 ± 13.22a	27.15 ± 12.09a	22.25 ± 2.90a
K‐exch (cmol/kg)	0.71 ± 0.22b	0.72 ± 0.15b	0.97 ± 0.06b	0.29 ± 0.00a	0.31 ± 0.16a	0.24 ± 0.01a
Mg‐exch (cmol/kg)	2.83 ± 0.13a	1.55 ± 0.04a	10.16 ± 3.44b	1.53 ± 0.21a	1.06 ± 0.13a	1.75 ± 0.00a

*Note:* Different letters following the mean values in the same row indicate significant differences among soil sampling sites according to Duncan′s test (at *p* < 0.05).

Abbreviations: AP, acacia plantation; BA, burnt area; OPP, oil palm plantation; RES, restored peat; RP, rubber plantation; SF, secondary forest.

### 3.2. Sequencing Output and Annotation Profile

**Figure 2:**
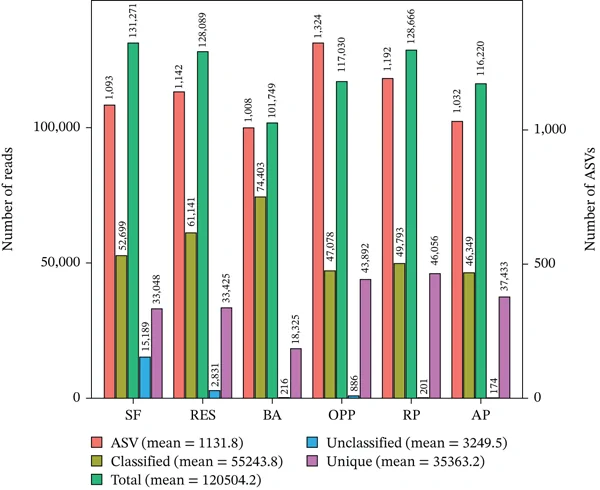
Number of annotated ASVs per site and ASV counts after quality filtering. ASVs (red) refers to the number of ASVs identified at each sampling site; classified (yellow‐green) refers to the number of ASVs designated with taxonomic annotation; total tags (green) refers to the total number of sequence reads that have undergone quality filtering; unclassified tags (blue) refers to the number of ASVs could not be taxonomically designated; unique tags (purple) refers to the number of tags that cannot be grouped into ASVs. Abbreviations: SF, secondary forest; RES, restored peat; BA, burnt area; OPP, oil palm plantation; RP, rubber plantation; and AP, acacia plantation.

The extracted DNA samples met the quality requirements for NGS analysis, with A260/280 ratios ranging from 1.81 to 1.89 and A260/230 ratios from 1.47 to 1.63. These values indicate low levels of coextracted inhibitors, which are suitable for PCR amplification and sequencing. All six composite samples produced high sequencing output and high annotation rates. On average, each site yielded 120,504 high‐quality sequence reads (total tags), of which 55,244 were assigned to classified ASVs. The mean numbers of unclassified tags and unique tags were 3250 and 35,363, respectively. In total, 94.4% of the sequences were successfully annotated, resulting in a classified‐to‐unclassified ratio of 17.0. The BA, RP, and AP sites exhibited the highest annotation rates (> 99%), followed by the RES site, with the SF site at 77.6%. Even though the OPP, RP, and AP sites generated fewer total tags, most sequences from these sites were successfully classified (Figure 2).

### 3.3. Pairwise Community Similarity Based on Jaccard Index

**Figure 3:**
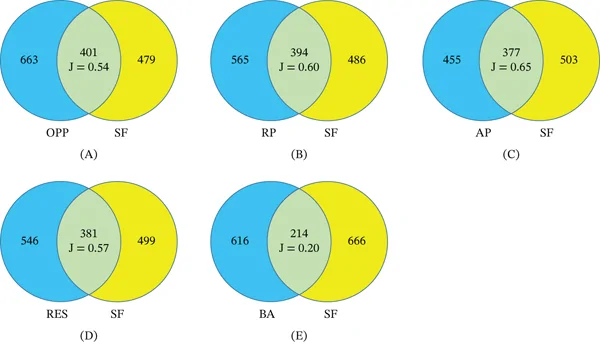
Number of ASVs unique to each site and shared between secondary forest (SF) and other sites: (A) oil palm plantation (OPP), (B) rubber plantation (RP), (C) acacia plantation (AP), (D) restored peat (RES), (E) and burnt area (BA). *J* values indicate pairwise community similarity based on the Jaccard index. Values reflect the extent of ASV overlap and site‐specific uniqueness across the landscape.

A Venn diagram and pairwise Jaccard index were used to assess overlap in ASV composition between individual sites (comparison between two communities). Jaccard index values ranged from 0.20 to 0.65 (Figure 3). Notably, the highest similarity occurred between AP–SF (0.65; Figure 3C) and RP–SF (0.60; Figure 3B). More than half of the ASVs present in each pair were shared. Sites RES–SF (0.57; Figure 3D) and OPP–SF (0.54; Figure 3A) showed moderate levels of similarity, implying that most ASVs in these pairs still overlapped. In contrast, site BA–SF (0.20; Figure 3E) had the lowest similarity, reflecting big compositional differences across BAs.

### 3.4. Shared and Site‐Specific ASVs Across Sites

**Figure 4:**
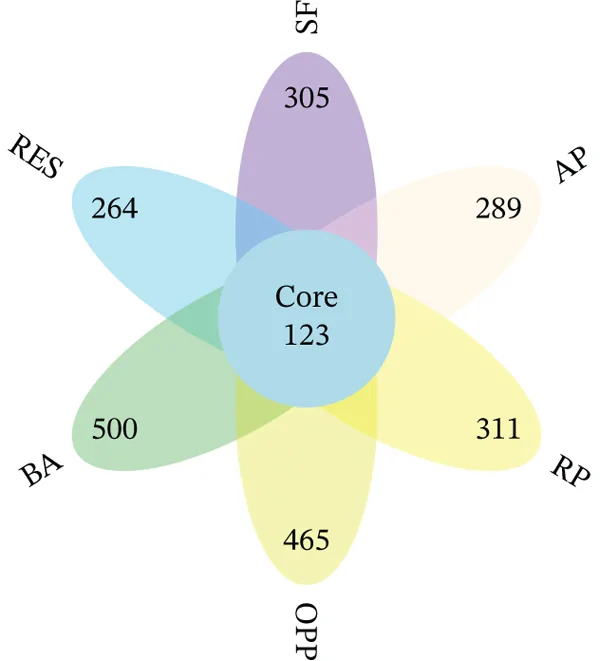
Number of ASVs unique to each site and those shared across sites. Values represent the extent of ASV overlap and site‐specific uniqueness across the landscape. Abbreviations: SF, secondary forest; RES, restored peat; BA, burnt area; OPP, oil palm plantation; RP, rubber plantation; and AP, acacia plantation.

In contrast to pairwise comparisons, the flower diagram highlights the limited overlap of ASVs across all sites. Only 123 ASVs were shared among the six sites (Figure 4), accounting for approximately 6% of the total ASVs and indicating a relatively small core community. The remaining ASVs (~94%) were unique to individual sites or restricted to subsets of sites. The higher number of unique ASVs observed at BA and OPP sites suggests stronger site‐specific filtering under fire and PL disturbance.

### 3.5. Relative Abundance of Bacteria at Phylum and Class Level

**Figure 5:**
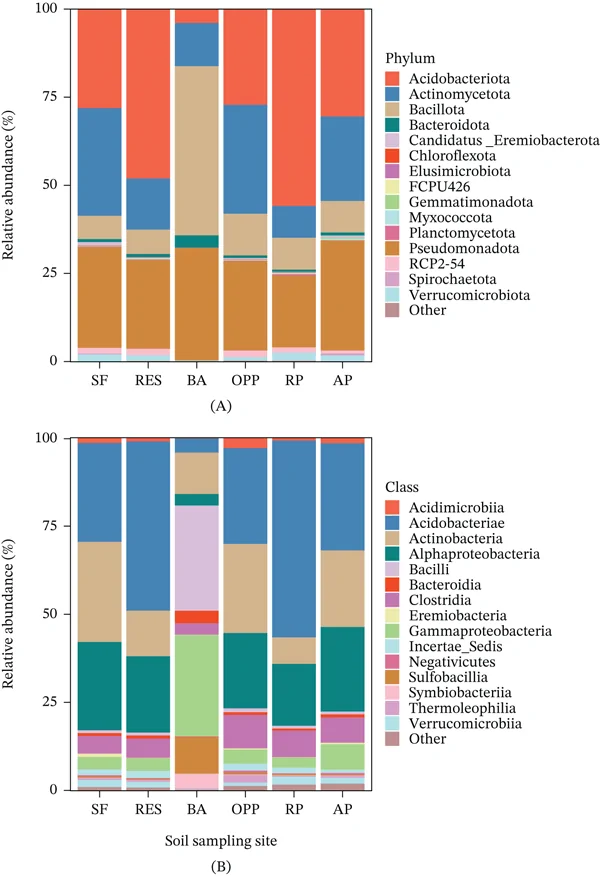
Dominant bacterial (A) phyla and (B) classes across land‐use types reveal conserved structure with site‐specific shifts. Abbreviations: SF, secondary forest; RES, restored peat; BA, burnt area; OPP, oil palm plantation; RP, rubber plantation; and AP, acacia plantation.

Acidobacteriota, Actinomycetota, and Pseudomonadota were the dominant bacterial phyla across the peat gradient (Figure 5A). This pattern appeared most pronounced in natural (SF) and restored (RES) peat and continued in PL‐converted sites (OPP, RP, and AP), where Acidobacteriota remained the predominant phylum. At the class level, Acidobacteriae, Actinobacteria, and Alphaproteobacteria continued to predominate (Figure 5B), demonstrating the persistence of a characteristic peat microbiome throughout the unburnt sites. Collectively, these three classes constituted over 50% of the total bacterial community. Conversely, the BA showed a pronounced compositional shift and manifested the most distinct bacterial community configuration. Bacillota (47.61%) became the predominant group at this site, and Bacilli and Gammaproteobacteria increased, whereas Acidobacteriota/Acidobacteriae decreased. These shifts at both the phylum and class levels distinguish the postfire bacterial community at BA from those at other sites. In contrast, the OPP PL site exhibited a distinct pattern, with Acidobacteriota, Actinomycetota, and Pseudomonadota showing relatively even representation at the phylum level, and Acidobacteriae, Actinobacteria, and Alphaproteobacteria appearing similarly balanced at the class level. This observation suggests that no single group dominated. Overall, the phylum‐ and class‐level profiles revealed three distinct configurations of bacterial community composition along the peat gradient: Acidobacteriota‐dominated communities were observed at intact, restored, and converted sites; Bacillota‐ and Bacilli‐dominated communities predominated at the BA; and the OPP site exhibited a relatively even distribution of phyla and classes.

### 3.6. Relative Abundance of Bacteria at Family and Genus Level

**Figure 6:**
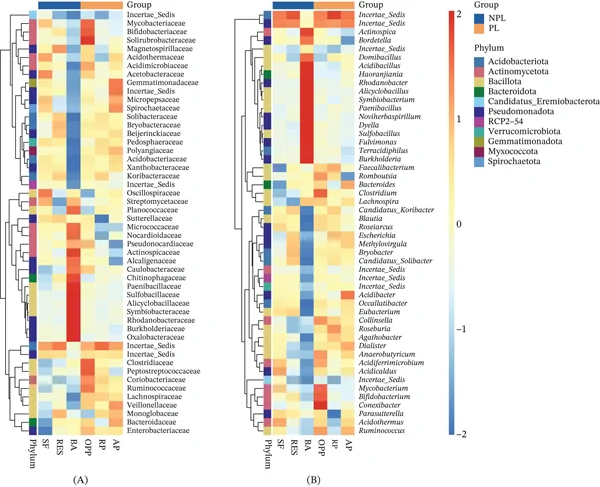
Heatmaps of dominant bacterial (A) families and (B) genera highlight taxonomic differentiation across sites. Abbreviations: SF, secondary forest; RES, restored peat; BA, burnt area; OPP, oil palm plantation; RP, rubber plantation; and AP, acacia plantation.

Heat‐map analysis of the 50 dominant taxa revealed three main compositional patterns across the peat gradient (Figure 6). Intact, restored, and PL peat (SF, RES, OPP, RP, and AP) communities were dominated by families and genera in the class Acidobacteriae. These included Acidobacteriaceae, Solibacteraceae, Bryobacteraceae, and Koribacteraceae (Figure 6A,B). Genera such as *Candidatus* Solibacter, *Candidatus* Koribacter, *Bryobacter*, *Acidicaldus*, *Acidiferrimicrobium*, and *Acidothermus* were recurrent, showing a persistent peat‐typical taxonomic core. In contrast, the BA had the most distinct configuration at both family and genus levels, with higher amounts of copiotrophic and disturbance‐associated taxa. These included Burkholderiaceae, Actinospicaceae, and Oxalobacteraceae at the family level, and *Dyella, Fulvimonas, Burkholderia, Rhodanobacter, Acidibacillus,* and *Paenibacillus* at the genus level. Many Acidobacteriae‐affiliated taxa declined at BA, and the Acidobacteriae incertae sedis group was less represented than at other sites. The PL site OPP showed a third distinct pattern, marked by the prominence of several non‐peat‐typical families such as Mycobacteriaceae, Bifidobacteriaceae, Solirubrobacteraceae, and Clostridiaceae, as well as their genera (*Mycobacterium*, *Bifidobacterium*, and *Clostridium*). These taxa had moderate relative abundances, unlike the extreme dominance at BA. In summary, family‐ and genus‐level heat‐map profiles identified a conserved Acidobacteriae‐centered core in nonburnt peat, a restructured assemblage at the BA, and a distinct PL community at OPP.

### 3.7. Alpha and Beta Bacterial Diversity Across Sites and Groups

Alpha and beta diversity analyses were performed using six composite sequencing samples, each representing one study site, generated by pooling four field subsamples. Consequently, statistical comparisons among individual sites were not possible, and analyses were therefore limited to comparisons between the PL and NPL groups, as described in Section 2. Alpha diversity analysis revealed variation in bacterial community diversity, richness, and evenness across the study sites (Table 3). Based on the Shannon index, the highest diversity was observed at the OPP site, followed by the SF and AP sites, indicating relatively higher bacterial diversity at these three sites. Conversely, the BA site exhibited the lowest Shannon diversity. The Simpson index also showed a similar pattern, with the highest values recorded at the RP, OPP, and SF sites, indicating a more even distribution of individuals among taxa. The ACE estimator indicated that the OPP site had the highest richness, followed by the RP and RES sites. Conversely, the lower ACE values at the AP and BA sites indicated relatively lower richness. Goods coverage values approaching 1 (> 0.999) across all sites suggest that the sequencing depth was sufficient to capture the entire bacterial diversity present in each site composite sample.

**Table 3:** Bacterial alpha diversity index.

Soil sampling site	Index			Goods_Coverage
	Shannon	Simpson	ACE	
SF	5.923	0.994	883.677	0.999
RES	5.772	0.990	931.382	0.999
BA	5.134	0.981	838.917	0.999
OPP	6.188	0.995	1068.015	0.999
RP	5.801	0.987	966.147	0.999
AP	5.830	0.992	835.814	0.999

Abbreviations: AP, acacia plantation; BA, burnt area; OPP, oil palm plantation; RES, restored peat; RP, rubber plantation; SF, secondary forest.

**Figure 7:**
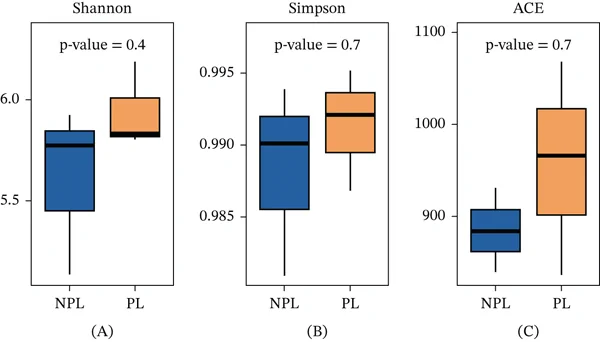
Alpha diversity indices between NPL (nonplantation) and PL (plantation) groups showing variation in bacterial diversity and richness of the peat microbiome: (A) Shannon diversity index, (B) Simpson diversity index, and (C) ACE richness estimator.

Comparison of alpha diversity indices between the NPL and PL groups showed that bacterial communities in both groups had comparable levels of diversity, richness, and evenness. The Shannon diversity showed no significant difference between the two groups (*p* = 0.4; Figure 7A). Simpson′s index showed a similar pattern (*p* = 0.7; Figure 7B), which means overall taxonomic evenness was similar in both groups. ACE estimates (*p* = 0.7; Figure 7C) also showed no significant difference in bacterial richness. Consistent with these patterns, the overall taxonomic composition of bacterial communities was broadly similar between the NPL and PL groups across different taxonomic levels (Figure S1). The dominant bacterial groups at the phylum level were Acidobacteriota, Actinomycetota, and Pseudomonadota, and at the class level, Acidobacteriae, Actinobacteria, and Alphaproteobacteria. There were some moderate differences in the frequency of these groups across sites. These results indicate that although community composition varied by site, the aggregated comparison between the NPL and PL groups revealed no clear shifts in dominant bacterial taxa.

**Figure 8:**
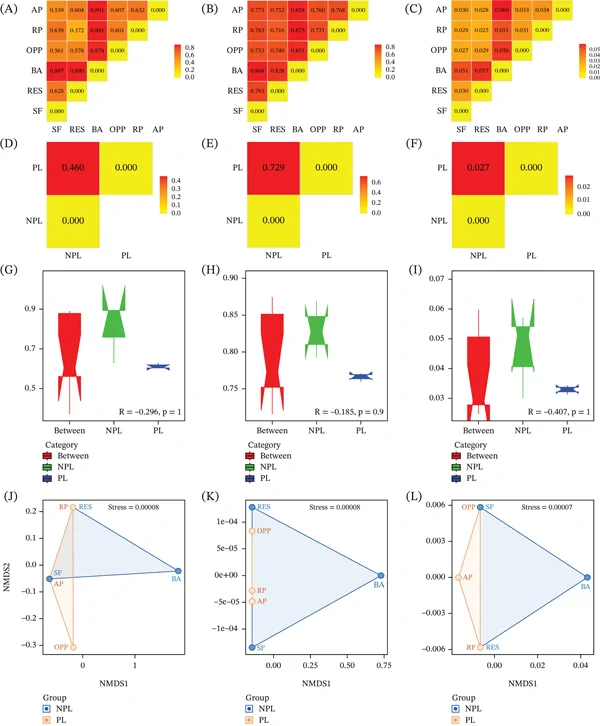
Beta diversity of peat bacterial communities across sites based on Bray–Curtis (A, D, G, and J), weighted UniFrac (B, E, H, and K), and unweighted UniFrac (C, F, I, and L) distance metrics. (A–C) Pairwise dissimilarity among sites. (D–F) Comparison of NPL and PL groups. (G–I) ANOSIM results for each metric. (J–L) NMDS ordinations derived from the corresponding distance matrices. Abbreviations: SF, secondary forest; RES, restored peat; BA, burnt area; OPP, oil palm plantation; RP, rubber plantation; AP, acacia plantation; NPL, nonplantation sites; PL, plantation sites.

Bacterial beta diversity, presented as a pairwise dissimilarity heat map, showed that most site pairs exhibit moderate‐to‐high community differences. Based on the Bray–Curtis distance (Figure 8A), intersite dissimilarity is generally high (0.53–0.90). The BA site is the least similar to all other sites (0.878–0.897). In contrast, the RES–RP pair showed the highest compositional similarity. Based on the unweighted UniFrac distance metric (Figure 8B), a similar pattern emerges, with dissimilarity values remaining high (0.71–0.87), indicating pronounced phylogenetic divergence among sites. The BA site again emerged as the most phylogenetically divergent (0.828–0.869). Based on the weighted UniFrac distance metric (Figure 8C), despite a narrower range (0.025–0.060), the relative patterns between sites remain consistent with those from the previous two metrics. The BA site shows the most remarkable dissimilarity among the sites (0.051–0.057). Meanwhile, the lowest values occur in pairs involving the SF and RES sites (0.025–0.030), indicating greater similarity when both relative abundance and phylogenetic relationships are considered.

Pairwise dissimilarity analysis between PL and NPL indicated moderate differences in bacterial community composition. The Bray–Curtis dissimilarity value of 0.460 (Figure 8D) suggests compositional differences between the groups, whereas the unweighted UniFrac (0.729; Figure 8E) and weighted UniFrac (0.027; Figure 8F) values indicate phylogenetic distances between bacterial communities in PL and NPL sites. However, multivariate community‐based statistical analysis did not support a significant separation between the groups. The ANOSIM results showed negative R values (–0.185 to –0.407; Figure 8G–I), indicating that variation within groups exceeded variation between the NPL and PL groups and suggesting that this grouping does not strongly structure bacterial community composition. Furthermore, the NMDS ordination showed very low stress values (0.00007–0.00008; Figure 8J–L). These near‐zero stress values likely reflect the limited number of composite samples and the relatively simple structure of the distance matrix, allowing the ordination to be represented with minimal distortion in two‐dimensional space. However, given that the ordination was based on only six composite samples, the near‐zero stress values should not be interpreted as strong evidence of a meaningful low‐dimensional community structure. Accordingly, the NMDS ordination should be regarded as an exploratory visualization and interpreted with appropriate caution. Consistent with this interpretation, the distribution of PL and NPL points did not show a clear pattern of separation, and although pairwise dissimilarity indicated differences in compositional and phylogenetic distances, multivariate analyses did not detect statistically significant differences between groups.

### 3.8. Correlation Between Soil Properties and Bacterial Diversity

**Figure 9:**
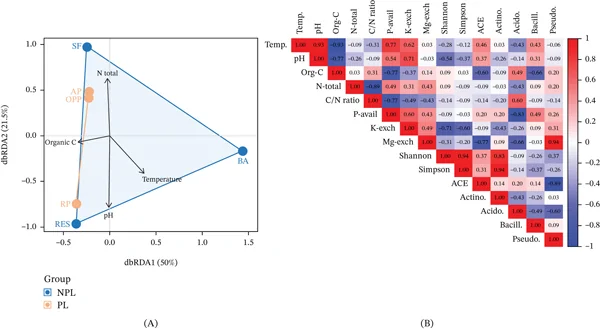
Correlation among soil physicochemical properties, bacterial diversity, and dominant bacterial phyla based on (A) distance‐based redundancy analysis (db‐RDA) and (B) Spearman correlation analysis. Abbreviations: Actino, relative abundance of Actinomycetota; Acido, relative abundance of Acidobacteriota; Bacill, relative abundance of Bacillota; Pseudo, relative abundance of Pseudomonadota.

The db‐RDA showed that the two main axes explained a significant proportion of the variation (db − RDA1 = 50*%* and db − RDA2 = 21.5*%*), indicating that ordination captured the main data gradient (Figure 9A). Site BA was separated in the positive direction of dbRDA1 and aligned with the temperature vector. In contrast, site SF was located in the upper‐left quadrant and associated with relatively higher N‐total values. In contrast, RES and RP were located in the lower left quadrant and associated with lower pH. Site OPP and AP were clustered closely together, indicating similar community compositions across the PL systems. Permutation tests showed that temperature (*F* = 3.71; *p* = 0.025) and pH (*F* = 3.65; *p* = 0.024) were significantly associated with bacterial community variation, whereas Org‐C showed a marginal association (*F* = 2.98; *p* = 0.049), and N‐total was not significant (*p* = 0.09). Although the model explains a high proportion of the variation (*R* ^2^ = 0.891), the lower corrected *R* ^2^ (0.453) suggests that some of the variation may be due to the limited sample size.

This pattern is consistent with the Spearman rank correlation results (Figure 9B). Temperature was positively correlated with pH (*ρ* = 0.93) and negatively correlated with Org‐C (*ρ* = −0.93). Soil pH showed a tendency toward a negative correlation with the Shannon index (*ρ* = −0.54; *p* = 0.27), and in the db‐RDA ordination, the direction was opposite to that observed for sites with relatively higher diversity (SF, OPP, and AP). Soil temperature also aligned with the BA site′s position on the db‐RDA1 axis. Org‐C showed a relatively weak relationship with diversity indices, although a moderate negative correlation with ACE was observed (*ρ* = −0.60; *p* = 0.21). Other variables, such as the C/N ratio, P‐avail, K‐exch, and Mg‐exch, showed weak relationships with diversity. At the phylum level, Pseudomonadota showed a positive correlation with Mg‐exch (*ρ* = 0.94; *p* = 0.005), whereas Acidobacteriota was positively correlated with C/N ratio (*ρ* = 0.60) and Org‐C (*ρ* = 0.49), but negatively with P‐avail (*ρ* = −0.83; *p* = 0.03) and Mg‐exch (*ρ* = −0.66). Bacillota showed a trend toward positive relationships with nutrient and pH variables, but a negative one with Org‐C, whereas Actinomycetota showed only weak relationships with environmental variables. Overall, the db‐RDA and Spearman correlation results suggest that variation in the bacterial community is likely related to environmental gradients, particularly temperature, pH, and nutrient availability. However, given the moderate corrected *R* ^2^ values and the predominance of insignificant correlations, these relationships should be interpreted with caution and are indicative only.

## 4. Discussion

### 4.1. Physicochemical Variations of Peat Soils Between Land‐Use Types

Peat soils across the study sites exhibited physicochemical characteristics typical of tropical peat ecosystems, including high Org‐C content and acidic pH. Variations in Org‐C, N‐total, P‐avail, K‐exch, and Mg‐exch contents indicate how land‐use change affects peat‐soil nutrient dynamics. Temperature remained relatively uniform across sites, as expected under relatively stable tropical climatic conditions, whereas other soil properties showed more pronounced variation (Table 2). Soil pH remained consistently acidic to extremely acidic across sites, reflecting the intrinsic properties of peat soils, where organic matter accumulation and the production of organic acids maintain low pH conditions [25, 61]. The soil remains acidic, even in peatlands that have been converted into rubber and oil PLs [62] or OPPs [63, 64].

Peatland conversion is often associated with reduced Org‐C [16, 25, 62], but relatively high Org‐C values were observed across both natural and converted sites in this study. This likely reflects the dominance of chemically stable carbon fractions in peat, such as humified and aromatic compounds that are resistant to decomposition [16, 17, 25]. PL vegetation also provides biomass inputs that help maintain surface carbon stocks [65, 66]. This combination of dominance of the stable carbon fraction and the supply of new biomass explains why Org‐C values in converted peatlands remain high [67, 68]. These observations are consistent with previous studies showing that a large proportion of peat carbon can persist even under disturbed conditions [18, 21, 69].

In contrast to carbon, nutrient availability showed clearer differentiation among land‐use types. Concentrations of P‐avail, K‐exch, and Mg‐exch were generally low, consistent with the oligotrophic nature of tropical peatlands, but were elevated at the BA site. The increases in P‐avail and Mg‐exch at the BA site are most likely attributable to the short‐term effects of fire, as sampling was conducted approximately 1 month after the fire. Peat fires can temporarily increase soil nutrient content and pH due to ash deposition [45, 70, 71]. Early vegetation regrowth after fire may also temporarily increase soil nutrient availability [72], although such changes rarely indicate long‐term improvement in soil fertility. Together, these physicochemical changes show a distinct gradient from intact oligotrophic conditions (the SF and RES sites) to more aerated and nutrient‐rich environments at converted sites (the BA, OPP, RP, and AP sites).

### 4.2. Annotation Efficiency and ASV Divergence

The classification pipeline achieved an overall annotation rate of 94.4%, indicating most ASVs could be assigned to known bacterial taxa using the SILVA reference database (Figure 2). The nearly complete (> 99%) annotation of the BA, RP, and AP sites suggests that these disturbed sites were dominated by taxa well represented in current reference databases, including copiotrophic groups commonly associated with converted or degraded peat [22, 73]. In contrast, the SF site, representing natural peat, exhibited the lowest annotation rate (77.6%), indicating a relatively larger proportion of ASVs that could not be classified using the current reference database. This pattern may reflect the limited representation of bacterial diversity from tropical peatlands in available genomic databases and highlights the need for further characterization of microbial communities in these ecosystems [74].

Analysis of ASV similarities and uniqueness further indicated that bacterial communities varied among land‐use types (Figure 3). Although each site had a distinct disturbance history, several pairs of sites still shared bacterial taxa, indicating the presence of a core peat microbiome tolerant of acidic and carbon‐rich environments. This pattern is consistent with the role of low pH and high organic matter as major environmental filters shaping microbial communities in tropical peat ecosystems [75, 76]. However, similarity among sites tended to decrease with increasing disturbance stress, such as fire (as in the BA site), which also contained a relatively large proportion of unique ASVs. Such shifts are consistent with previous studies showing that fire and land‐use change can alter soil bacterial community composition and generate distinct microbial assemblages [77]. Overall, only about 6% of ASVs were shared across sites (Figure 4), indicating high diversity of bacterial communities in tropical peatland landscapes and high levels of community uniqueness across land‐use types. The occurrence of numerous site‐specific ASVs suggests that different land‐use types harbor distinct bacterial assemblages, although the ecological roles of these unique taxa require further investigation [22, 73, 78].

### 4.3. Land‐Use and Disturbance‐Induced Shifts in Core Peat Bacterial Communities

Land‐use change in tropical peatlands was associated primarily with shifts in the relative abundance of dominant bacterial groups rather than clear changes in overall bacterial community composition (Figures 6 and 7). The phyla Acidobacteriota, Pseudomonadota, and Actinomycetota were consistently dominant across all sites, indicating a broadly conserved bacterial community despite contrasting land‐use types [29, 35, 79]. However, multivariate analysis did not detect significant differences among land‐use types, limiting the strength of inference regarding community separation. The consistent dominance of Acidobacteriota across both intact and converted peatlands aligns with previous studies [22, 80, 81], which highlight their adaptation to acidic, carbon‐rich, and oxygen‐limited environments [35]. Moreover, this finding aligns with earlier work showing the dominance of oligotrophic and acidophilic bacterial groups across various peatland types, albeit with varying relative abundances [26, 28]. In contrast, the relatively higher abundance of Actinomycetota and Pseudomonadota in PL sites is consistent with patterns reported in other PL systems [11, 75, 81] and may be associated with shifts in vegetation‐derived carbon inputs and improved soil aeration that increase the availability of more labile organic substrates [23, 82].

An additional feature of the OPP site was the presence of several bacterial families not commonly reported in peatland bacterial communities, including Mycobacteriaceae, Bifidobacteriaceae, and Clostridiaceae. Previous studies of both tropical and temperate peatlands consistently describe peat soil microbiomes as being dominated by acidophilic and oligotrophic bacterial groups, particularly members of the phyla Acidobacteriota, Pseudomonadota, and Actinomycetota [22, 75, 83, 84]. In contrast, members of Bifidobacteriaceae are predominantly associated with the gastrointestinal tracts of humans and other mammals and are only occasionally detected in soil environments, where their occurrence is generally attributed to external inputs rather than indigenous soil populations [85–87]. Similarly, members of Mycobacteriaceae and Clostridiaceae occur in soils, sediments, animal wastes, and aquatic environments, although some taxa can persist under anaerobic conditions [88, 89]. Their exclusive occurrence at the OPP site may therefore reflect localized inputs associated with wildlife activity, agricultural inputs (e.g., animal manure), or anthropogenic activities related to PL management, rather than typical members of peat bacterial communities [11]. However, because this study was based on a single composite sample per site and taxonomic assignments derived from 16S rRNA gene sequencing, these potential sources cannot be distinguished. Consequently, the ecological significance and origin of these bacterial families remain uncertain and warrant further investigation using replicated sampling and complementary approaches, such as shotgun metagenomic sequencing.

Site BA exhibited the most distinct community patterns. Peat fires at this site altered environmental conditions differently from the other land‐use types, including increased pH, nutrient availability, and more readily degradable carbon [90]. These changes were associated with an increased relative abundance of Bacillota [91]. This pattern is consistent with previous observations that postfire environments tend to favor disturbance‐tolerant and metabolically flexible taxa, particularly spore‐forming bacteria capable of surviving heat stress and rapidly recolonizing disturbed soils [80, 92, 93]. Some genera within this group, such as *Paenibacillus*, *Acidibacillus*, and *Clostridium*, exhibit metabolic flexibility, enabling them to utilize more labile carbon sources [94, 95]. In contrast, the relative abundance of Acidobacteriota and some of its lineages tended to decrease at site BA, likely related to changes in pH, increased nutrient availability, and more oxygenated conditions, which generally favor oligotrophic groups less [96]. However, responses across these groups can vary by subgroup [97]. Furthermore, increases in the relative abundance of copiotrophic groups such as Actinobacteria, Gammaproteobacteria, and Betaproteobacteria were also observed, which have previously been reported to be associated with nutrient‐ and dissolved carbon‐rich postfire peat soils [91, 98–100].

Vegetation differences among land‐use types may also contribute to the observed variation in bacterial community composition [79, 101] by altering litter input, root exudates, and the quantity and quality of Org‐C entering the peat soil. Compared with SFs and restored peatlands, PL systems generally receive greater inputs of relatively labile organic substrates [17, 102]. Such differences in substrate quality are likely associated with a greater relative abundance of copiotrophic bacterial groups, whereas forested peatlands continue to support bacterial taxa adapted to more recalcitrant organic matter and nutrient‐limited conditions [27, 34, 103]. Despite these patterns, approximately 6% of ASVs were shared across all sites, indicating the persistence of a conserved peat‐associated bacterial community. This suggests that, under the physicochemical constraints typical of peat soils, many bacterial taxa can persist across land‐use types, with variation expressed primarily as changes in relative bacterial abundance rather than complete community replacement [22, 78]. These observations provide a broader ecological context for understanding how persistent environmental filtering may contribute to the long‐term stability of peat bacterial communities despite contrasting land‐use histories, a mechanism discussed further in the following section.

### 4.4. Environmental Filtering and the Persistence of a Conserved Peat Microbiome

Across the six peatland sites, soil conditions were consistently characterized by high Org‐C content, strongly acidic pH, and variable nutrient availability, with elevated P‐avail, K‐exch, and Mg‐exch observed at converted sites. These physicochemical conditions define a shared environmental setting in which bacterial communities develop [104–106]. Within this context, persistently low pH, high Org‐C, and waterlogged conditions, together with the accumulation of phenolic compounds that suppress extracellular enzyme activity and slow decomposition, a mechanism known as the enzymatic latch [107–109], likely constrain the range of microbial taxa able to persist [110]. These constraints are consistent with the widespread dominance of taxa such as Acidobacteriota across both intact and converted sites, reflecting adaptation to acidic, carbon‐rich, and oxygen‐limited environments [22, 80, 81].

At the same time, variation in nutrient availability at disturbed or converted sites was associated with shifts in the relative abundance of bacterial groups. For example, increased P‐avail, K‐exch, and Mg‐exch at the BA site coincided with higher relative abundance of copiotrophic taxa such as Bacillota [94, 95], whereas beta‐diversity patterns suggested partial differentiation of this site in ordination space. Taken together, these patterns indicate that peat bacterial communities are organized within a relatively constrained environmental framework, where long‐standing peat physicochemical conditions limit the range of taxa that can persist. Under these conditions, land‐use change appears to be associated primarily with shifts in the relative abundance of dominant taxa rather than complete replacement of bacterial communities. The presence of a small but consistent fraction of shared ASVs across sites further supports the existence of a conserved core microbiome adapted to peat environments [83, 111].

However, these patterns should be interpreted cautiously. Multivariate analyses did not detect statistically significant differences among land‐use types, the db‐RDA model was not significant, and most Spearman correlations were weak (*p* > 0.05), indicating that variation within groups exceeded differences between groups. Consequently, the observed relationships between bacterial communities and environmental variables should be interpreted as indicative rather than definitive, and apparent differences in ordination space should be viewed with caution. In addition, changes in bacterial taxonomic composition do not necessarily correspond to alterations in ecosystem function or carbon stability [112, 113]. Deforested and drained/degraded tropical peatlands have been shown to lose substantial amounts of stored carbon through runoff and fluvial pathways, highlighting the long‐term vulnerability of these ecosystems to land‐use change [114–116]. However, the present taxonomic dataset alone cannot directly demonstrate altered ecosystem processes or carbon dynamics. Given the limited number of sites and the lack of replication at the land‐use level, this study should therefore be considered exploratory and hypothesis‐generating.

## 5. Conclusions

This study shows that land‐use change and fire in tropical peatlands are associated with shifts in the relative abundance of bacterial taxa, rather than a complete restructuring of community composition. Major phyla such as Acidobacteriota, Actinomycetota, and Pseudomonadota remained consistently present across sites, indicating the persistence of a shared community framework under varying land‐use conditions. In contrast, the burned site was characterized by a marked increase in Bacillota, which belongs to the group of copiotrophic taxa, alongside a relative decline in several oligotrophic lineages typically associated with intact peat environments. Variations in soil properties, particularly pH and nutrient availability (P‐avail, K‐exch, and Mg‐exch), were associated with differences in community composition and diversity. The combination of a small shared ASV fraction and high beta diversity suggests that peat bacterial communities are spatially heterogeneous, with local environmental conditions shaping relative abundance patterns within a broadly conserved taxonomic structure.

Overall, these findings support a model in which persistent physicochemical constraints structure tropical peat microbiomes yet remain responsive to disturbance through compositional adjustments rather than complete turnover. However, given the limited replication and single‐time‐point sampling, as well as the lack of consistently strong statistical support for relationships between environment and community, these findings should be interpreted as exploratory and hypothesis‐generating rather than definitive. Further research incorporating greater spatial replication, temporal dynamics, and functional analyses will be essential for better understanding how changes in microbial communities relate to ecosystem processes and long‐term peatland stability.

## Author Contributions

D.Z. conceived the idea and designed the research. D.Z., P.K.Z., F.A.S.S., and N. performed the experiments and analyzed the data. D.Z., P.K.Z., and F.A.S.S. drafted the manuscript. D.Z. and N. reviewed and provided feedback. D.Z. revised and finalized the manuscript.

## Funding

This work was supported by the Directorate of Resources, Directorate General of Higher Education, Research, and Technology, Ministry of Education, Culture, Research, and Technology, Indonesia, through the Higher Education Excellence Basic Research Program (PDUPT), under contract number 1416/UN19.5.1.3/PT.01.03/2021.

## Disclosure

All authors reviewed and approved the final published version of the manuscript.

## Ethics Statement

This article does not contain any studies involving human or animal participants. Peat soil sampling and analysis were conducted by ethical scientific procedures and without any negative impact on the environment, as well as by general research guidelines applicable at the University of Riau.

## Conflicts of Interest

The authors declare no conflicts of interest.

## Supporting information


**Supporting Information** Additional supporting information can be found online in the Supporting Information section. Supporting Information. Figure S1: A comparison of bacterial community composition between plantation (PL) and nonplantation (NPL) peatlands at the phylum, class, family, and genus levels. The figure provides additional taxonomic detail supporting the comparison of bacterial community profiles between the two land‐use groups.

## Data Availability

The raw 16S rRNA gene amplicon sequencing data generated in this study have been deposited in the NCBI Sequence Read Archive (SRA) under BioProject accession number PRJNA1463193. The corresponding BioSample accessions are SAMN59294429, SAMN59294430, SAMN59294431, SAMN59294432, SAMN59294433, and SAMN59294434.
