# Supplementary material for: Shifts in Soil Bacterial Diversity and Abundance at Various Taxonomic Levels in Tropical Peatlands Under Land‐Use Change
Source: Int J Microbiol. 2026 Aug 29;2026:3336189. doi: 10.1155/ijm/3336189 (PMC13525552; doi:10.1155/ijm/3336189)
Supplement: Supplementary file 1 [file IJM-2026-3336189-s001.docx]

FIGURE S1: Comparison of bacterial community composition between plantation (PL) and non-plantation (NPL) peatlands at (A) phyla level, (B) classes level, (C) families level, and (D) genera level.
